# Surgical regenerative methods for peri-implantitis treatment: A systematic review and meta-analysis

**DOI:** 10.34172/japid.2024.013

**Published:** 2024-07-31

**Authors:** Soheil Shahbazi, Saharnaz Esmaeili, Armin Shirvani, Reza Amid, Mahdi Kadkhodazadeh

**Affiliations:** ^1^Dentofacial Deformities Research Center, Research Institute of Dental Sciences, Shahid Beheshti University of Medical Sciences, Tehran, Iran; ^2^Iranian Center for Endodontic Research, Research Institute of Dental Sciences, Dental School, Shahid Beheshti University of Medical Sciences, Tehran, Iran; ^3^Dental Research Center, Research Institute of Dental Sciences, Shahid Beheshti University of Medical Sciences, Tehran, Iran; ^4^Department of Periodontics, Shahid Beheshti University of Medical Sciences, Tehran, Iran

**Keywords:** Bone regeneration, Bone substitutes, Peri-implantitis, Regeneration, Regenerative medicine

## Abstract

**Background.:**

The purpose of this study was to review the literature on the efficacy of different surgical regenerative methods for peri-implantitis treatment.

**Methods.:**

A preliminary search was conducted in seven electronic databases. The studies included in the analysis implemented surgical regenerative treatment in at least one study group. Baseline and follow-up values for bleeding on probing (BoP), pocket depth (PD), plaque index (PI), bone level (BL), and bone gain (BG) were extracted. The standardized mean difference (SMD) was calculated using Cohen’s d or Hedges’ g, and a random-effects-restricted maximum likelihood (REML) method was applied for the meta-analysis.

**Results.:**

Fifteen studies were included in the qualitative synthesis. The meta-analysis was performed on six studies comparing regenerative techniques that involved bone grafts with those that did not. The overall effect size for using bone grafts at the one-year follow-up was 0.04 (95% CI: -0.26‒0.35; *P*=0.78) for BoP, -0.08 (95% CI: -0.42‒0.27; *P*=0.66) for PD, 0.37 (95% CI: 0.08‒0.65; *P*=0.01) for PI, -0.44 (95% CI: -0.84 to -0.03; *P*=0.03) for BL, and 0.16 (95% CI: -0.68‒1.01; *P*=0.70) for BG.

**Conclusion.:**

Various materials have been employed for peri-implant defect filling and coverage. A bone substitute did not significantly improve BoP, PD, and BG values, while PI and BL were significantly ameliorated at one-year follow-up. However, recommending a single unified protocol as the most effective for surgical regenerative treatment of peri-implantitis was not feasible.

## Introduction

**Figure 1:**
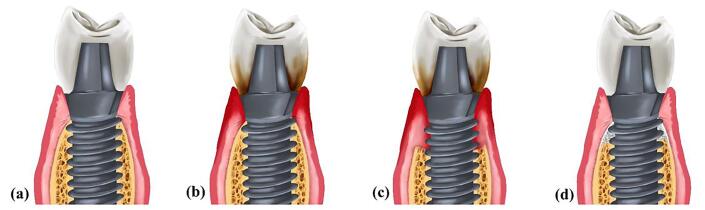
(a) Healthy peri-implant tissues. (b) Peri-implant mucositis. The accumulation of plaque has led to soft tissue inflammation. (c) Peri-implantitis. Inflammation has invaded both soft and hard tissues. Therefore, bone loss is evident. (d) A regenerated site

 Despite the evidence showcasing the high success rate of dental implants in oral rehabilitation, healthcare providers must be aware of possible complications after implant placement.^1,2^ Peri-implant mucositis affects the soft tissue surrounding implants and can progress into peri-implantitis if left untreated. Peri-implantitis is characterized by bleeding on probing (BoP) and/or suppuration, increased probing depth and/or marginal recession alongside progressive radiographic bone loss compared to previous visits (Figure 1).^3^ The average prevalence rate of peri-implantitis is 22%, with a range of 1%‒47%.^4^

 The incidence of peri-implantitis is associated with the accumulation of bacterial plaque primarily consisting of the microorganisms involved in periodontitis.^3,5^ However, *Porphyromonas gingivalis*, *Tannerella forsythia*, and *Treponema denticola* are found at higher concentrations in samples obtained from peri-implantitis patients.^6^ Factors such as smoking, untreated periodontitis, irregular maintenance, and diabetes mellitus have been identified as risk factors for peri-implantitis.^7-10^ Local factors such as excess cement, incorrect prosthesis seating, implant malpositioning, implant micro- and macro-design, abutment connection type, and excessive mechanical loads can all contribute to disease progression.^5,11^

 Peri-implantitis treatments primarily aim to eradicate tissue inflammation, stop disease progression and bone loss, regenerate lost supportive tissues, and restore osseointegration.^12^ These treatments encompass diverse surgical and non-surgical approaches, such as mechanical debridement, application of antiseptics, antibiotic therapy, surgical flaps, and resective or regenerative surgeries.^13-19^ Resective surgery is ideal for shallow defects, while deeper intrabony defects are better suited to regenerative strategies.^20,21^ A regenerative approach would be preferred if the bony defect has a minimum depth of 3 mm, is enclosed by three or four walls, and sufficient keratinized mucosa is present.^22^ In cases where a failing implant is predicted to have a poor prognosis or the aforementioned treatment strategies do not lead to success, explantation is the inevitable choice.^23^

 The regenerative approach involves surface decontamination and the use of bone grafts with or without a barrier membrane.^24^ When selecting a treatment strategy, decisions must be made concerning the decontamination method, graft material, barrier membrane, and surgical technique. Decontamination can be achieved through mechanical or chemical methods, such as using acids, antiseptics, abrasives, and lasers.^25-28^ Moreover, different graft materials can be used, including allografts, autografts, xenografts, etc.^28,29^ If necessary, a range of resorbable or non-resorbable membranes can be used to cover the graft.^30^ Given the multiple biomaterials and techniques reported in the literature, numerous protocols can be used to manage peri-implantitis.^24,31^ However, the consensus is that no specific biomaterial or treatment protocol for peri-implantitis has proven superior to others.^32^

 Hence, this systematic review aimed to compare various surgical regenerative interventions for peri-implantitis management based on their clinical and radiographic enhancements.

## Methods

###  Methodology and protocol registration

 The study adhered to PRISMA (Preferred Reporting Items for Systematic Reviews and Meta-analyses) guidelines,^33^ and the protocol was registered in PROSPERO (International Prospective Register of Systematic Reviews) under the ID CRD42021288572.

###  Focused question

 When treating peri-implantitis patients, which surgical regenerative protocols lead to more significant improvements in clinical and radiographic parameters during a minimum follow-up duration of 12 months?

###  Eligibility criteria

 A PICO-style search strategy was designed as follows:

- Population (P): Individuals aged ≥ 18 diagnosed with peri-implantitis without a systemic health condition that would contraindicate surgical treatments.
- Intervention (I): Surgical regenerative treatment of peri-implantitis in at least one study group
- Comparison (C): Comparing different surgical regenerative treatments
- Outcome (O): Changes in clinical and radiographic parameters such as BoP, probing depth (PD), plaque index (PI), bone level (BL), and bone gain (BG)

Only randomized and non-randomized clinical trials meeting the following criteria were included:

-  ≥ 12 months follow-up
- A minimum sample size of 10 implants per study

The exclusion criteria were as follows:

- Review articles, case reports/series, and abstracts
- Animal and in vitro studies
- Studies investigating retrograde peri-implantitis
- Studies exhibiting a high risk of bias
- Studies not mentioning the disease definition or providing an unclear definition

###  Search strategy

 In January 2022, the initial search was performed in electronic databases, including PubMed, Embase, Web of Science, Scopus, Google Scholar, Cochrane CENTRAL, and ProQuest (for grey literature). An updated search was also conducted in May 2022. The search terms used in these electronic databases included the following: ((peri-implantitis) OR (peri-implant disease) OR (peri-implant disease)) AND ((regenerative medicine) OR (biomaterial) OR (regenerative surgery) OR (surgical regeneration) OR (bone graft) OR (bone substitute) OR (membrane) OR (growth factor)) AND ((treatment) OR (management) OR (therapy)).

 Notably, the search query was tailored to the search guidelines of each database. The reference lists of the included studies were also reviewed to uncover any pertinent studies that might have been overlooked. Additionally, a manual search was conducted in journals related to dental implants and peri-implant diseases to identify any articles that might have been missed in the electronic search.

###  Screening and data extraction

 The results were imported into EndNote X20 software (Clarivate Company, Philadelphia, USA), and the duplicates were removed. The titles and abstracts of the remaining articles were independently screened by two reviewers who were unaware of each other’s decisions. After omitting irrelevant results, the full texts of the remaining articles were meticulously read and compared against the inclusion and exclusion criteria. In case of disagreements between the two reviewers, a third reviewer was consulted to reach an agreement.

 The data extraction was limited to the following items:

- Author and year: The name of the first author and publication year
- Sample size: The number of patients/implants included in the study
- Follow-up period: The period during which clinical and radiographic parameters changed
- Clinical and radiographic parameters, including BoP, PD, PI, BL, and BG
- Decontamination: The actions taken to detoxify the implant surface and the methods for debridement and removal of granulation tissue
- Bone graft: The type of bone graft used to fill the intraosseous defect with the aim of regeneration (e.g., autograft, xenograft, etc.)
- Membrane: The type of membrane used to cover the bone substitute, if used (e.g., collagen membrane)
- Postoperative care: Prescribed agents to decrease the risk of infection at the surgery site (e.g., chlorhexidine (CHX), antibiotics, etc.)
- Merging status: The status of the implants after the treatment, specifically whether they were submerged or non-submerged
- Complications: Complications such as infection, membrane exposure, etc., and any loss of samples
- Conclusion: A summary of the findings

###  Risk of bias assessment

 Two authors independently performed the risk of bias assessment concurrent with data extraction. The Cochrane risk of bias tool for randomized clinical trials was used.^34^ The tool evaluates bias across five distinct domains: randomization process, deviations from intended interventions, missing outcome data, outcome measurement, and selection of reported results. A study was deemed to have a “low risk of bias” if all domains displayed a low risk. Conversely, the presence of high risk in even one domain classified the study as having a “high risk of bias.” If a study presented some concerns in at least one domain but did not manifest a high risk in any domain, it was categorized as having “some concerns.”

###  Data analysis

 The standardized mean difference (SMD) was computed for each outcome measure (BoP, PD, PI, BL, and BG) using Cohen’s d or Hedges’ g. Meta-analysis was performed using the random-effects restricted maximum likelihood (REML) method in Stata version 17 (Stata Corp., College Station, Texas, USA). Potential sources were examined through meta-regression analysis to assess the presence of heterogeneity.

## Results

###  Study selection

**Figure 2:**
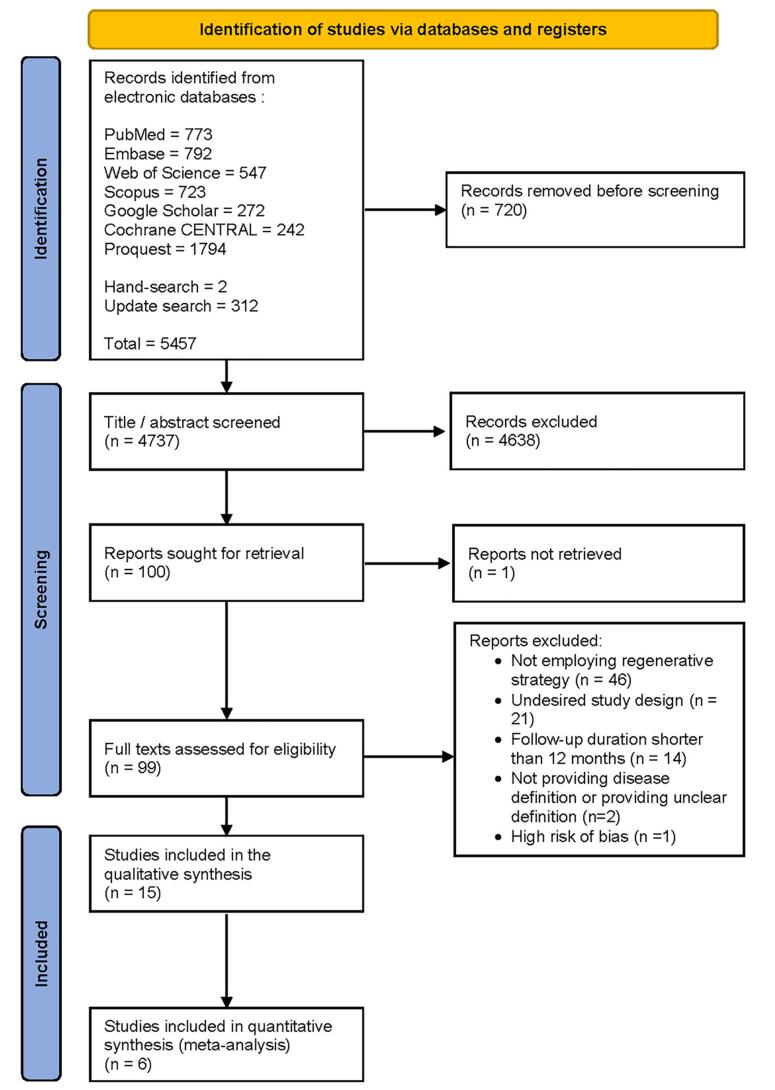
The preferred reporting items for systematic reviews and meta-analyses (PRISMA) flow diagram demonstrating the study selection process

 The initial search in electronic databases, hand-search, and update search yielded 5457 results, which were reduced to 4737 after deduplication. Further screening of titles and abstracts led to the exclusion of 4638 studies, leaving 99 potentially relevant studies. After a thorough examination of the full texts, 15 studies were chosen for data extraction (Figure 2). The remaining 84 studies were excluded for various reasons, such as not providing a disease definition or providing an unclear definition, a follow-up duration < 12 months, an undesired study design, not employing a regenerative strategy, and exhibiting a high risk of bias.

###  Study characteristics

 The 15 remaining studies included 12 original randomized clinical trials and three long-term follow-up studies from these original trials. The total number of patients who received treatments for peri-implantitis was 455 (507 implants), with a mean age of 63.13 ± 10.72 years (ranging from 54.4 to 73.5 years). The examined implants were in function for 7.06 ± 3.08 years on average (ranging from 4.82 to 14 years). Ten studies included smoking patients,^29,35-43^ one excluded smokers,^44^ and one did not report smoking status.^45^ Smokers comprised 31.85% of the participants in studies reporting smoking status (ranging from 15% to 69.6%).

###  Outcome measures

 BoP was reported in 11 studies,^29,35-43,45^ and the initial measurements showed a minimum of 15.4% and a maximum of 100%. The pre- and postoperative PDs were measured in 12 studies.^29,35-45^ The baseline PD ranged from 4.9 mm to 7.6 mm. PI was reported in 10 studies, but two different indices were used. Seven studies used the O’Leary index,^29,35,38-40,42,43^ and three used Silness & Löe.^36,37,45^ The baseline measurements showed a minimum of 13% and a maximum of 45% through the former index and a minimum of 0.5 and a maximum of 1.21 through the latter. In three out of seven studies reporting BL, the implant shoulder was considered the coronal reference point,^38,40,44^ and four studies did not clarify their reference points.^35,39,42,43^ The baseline BL ranged from 3.91 mm to 5.3 mm in the first group and 3.6 mm to 5.6 mm in the second group. The reports of 11 studies evaluating BG showed a minimum value of 0.2 mm^29,42^ and a maximum of 3.58 mm.^35^ However, some interventions led to bone loss, with a maximum loss of 1.9 mm in a study by Andersen et al (Tables 1 and 2).^41^ Six studies included clinical and radiographic measurements for follow-up periods exceeding one year (Table 3).

**Table 1:** Summary of baseline and one-year measurements reported in the studies

**Author & Year**	**Study Design**	**Sample Size**	**Follow-Up **	**Clinical & Radiographic Parameters**								
				**BoP**		**PD**		**PI**		**BL**		**BG**
				**Baseline**	**1 year**	**Baseline**	**1 year**	**Baseline**	**1 year**	**Baseline**	**1 year**	**1 year**
Renvert et al (2021)^43^	RCT	Total: 71 Patients/71 Implants G1: 37 Patients/37 Implants G2: 34 Patients/34 Implants	12 m	G1: 15.9% ± 19 G2: 15.4% ± 15.4	G1: 9.7% ± 11.7 G2: 8.3% ± 9.3	G1: 6.7 ± 1.5 G2: 6.8 ± 1.3	G1: 4.8 ± 1.5 G2: 4.5 ± 1.5	G1: 23.8% ± 23 G2: 27.3% ± 23.1	G1: 16.5% ± 16.7 G2: 14.6% ± 12.9	G1: 4.4 ± 1.8 G2: 4.9 ± 1.8	G1: 2.1 ± 1.6 G2: 3.6 ± 2.3	G1: 2.3 ± 1.2 G2: 1.1 ± 1.1
Emanuel et al (2020)^44^	RCT	Total: 27 Patients/32 Implants G1: 14 Patients/18 Implants G2: 13 Patients/14 Implants	12 m	NM	NM	G1: 6.76 ± 1.74 G2: 6.39 ± 1.78	G1:4.36 ± 1.41 G2: 5.43 ± 1.92	NM	NM	G1: 4.78 ± 1.58 G2: 4.55 ± 1.97	G1:3.9 ± 1.45 G2:4.88 ± 2.11	G1:0.88 ± 1.23 G2:-0.33 ± 1
Polymeri et al (2020)^38^	RCT	Total: 24 Patients/24 Implants G1: 11 Patients/11 Implants G2: 13 Patients/13 Implants	12 m	G1: 100% G2: 100%	G1: 45.5% ± 33.2 G2: 50% ± 10.2	G1:7 ± 1.8 G2:7.1 ± 1.2	G1: 3.4 ± 0.6 G2: 3.4 ± 0.5	G1: 31.7% ± 13.1 G2: 29.4 ± 13	G1: 17.5% ± 11.5 G2: 14% ± 9.3	G1: 5.3 ± 1.2 G2: 4.9 ± 1.1	G1: 3.1 ± 1.3 G2: 2.1 ± 1.3	G1: 2.2 ± 0.8 G2: 2.8 ± 1.3
De Tapia et al (2019)^40^	RCT	Total: 30 Patients/30 Implants G1: 15 Patients/15 Implants G2: 15 Patients/15 Implants	12 m	G1: 100% G2: 100%	G1: 20% ± 41 G2: 46% ± 52	G1: 6.16 ± 1.27 G2: 6.17 ± 0.98	G1: 3.31 ± 0.72 G2: 3.87 ± 0.81	G1: 14.54% ± 6.12 G2: 18.34% ± 6.54	G1: 16.56% ± 8.39 G2: 18.78% ± 5.9	G1: 3.91 ± 0.93 G2: 4.15 ± 0.84	G1: 1.2 ± 1.14 G2: 2.65 ± 1.44	G1: 2.51 ± 1.21 G2: 0.73 ± 1.26
Isler et al (2018)^37^	RCT	Total: 52 Patients/52 Implants G1: 26 Patients/26 Implants G2: 26 Patients/26 Implants	12 m	G1: 97.12% ± 10.79 G2: 97.12% ± 8.15	G1: 35.58% ± 30.14 G2: 29.81% ± 30.02	G1: 5.92 ± 1.26 G2: 5.41 ± 1.16	G1: 3.71 ± 1.09 G2: 2.7 ± 0.8	G1: 0.96 ± 0.58 * G2: 1.12 ± 0.41 *	G1: 0.67 ± 0.35 G2: 0.45 ± 0.44	NM	NM	G1: 1.63 ± 1 G2: 1.98 ± 0.75
Isler et al (2018)^36^	RCT	Total: 41 Patients/60 Implants G1: 20 Patients/30 Implants G2: 21 Patients/30 Implants	12 m	G1: 96.6% ± 10.85 G2: 97.5% ± 10.06	G1: 15.8% ± 19.1 G2:25% ± 21.7	G1: 6.27 ± 1.42 G2: 5.73 ± 1.11	G1: 2.75 ± 0.7 G2: 3.43 ± 0.85	G1: 1.21 ± 0.57 * G2: 0.96 ± 0.63 *	G1: 0.22 ± 0.17 G2: 0.49 ± 0.27	NM	NM	G1: 2.32 ± 1.28 G2: 1.17 ± 0.77
Renvert et al (2018)^42^	RCT	Total: 41 Patients/41 Implants G1: 21 Patients/21 Implants G2: 20 Patients/20 Implants	12 m	G1: 100% G2: 100%	G1:52.4% G2:65%	G1: 6.6 ± 1.8 G2:6 ± 1.7	G1: 2.6 ± 1.5 G2: 3.9 ± 2.7	G1: 30% G2: 45%	G1: 10% G2: 25%	G1: 3.6 ± 1 G2: 3.7 ± 2	G1: 2.9 ± 1.2 G2: 3.1 ± 1.2	G1: 0.7 ± 0.9 G2: 0.2 ± 0.6
Andersen et al (2017) ^41^	RCT	Total: 12 Patients/12 Implants G1: 6 Patients/6 Implants G2: 6 Patients/6 Implants	7 y	G1: 92% G2: 100%	G1:77% G2:83%	G1:6.5 ± 1.9 G2:6.5 ± 2.3	G1: 4.9 ± 1.8 G2: 4.4 ± 4.4	NM	NM	NM	NM	G1: -1.9 ± 2 G2: -1.3 ± 1.4
Isehed et al (2016)^39^	RCT	Total: 29 Patients/29 Implants G1: 15 Patients/15 Implants G2: 14 Patients/14 Implants	5 y	G1: 93.3% G2: 85.7%	G1: 70% G2:70%	G1: 6.5 † G2: 7.6 †	NM	G1: 23% † G2: 15% †	G1: 3% † G2: 0% †	G1: 5.6 † G2: 4.2 †	NM	G1: 0.9 † G2: -0.1 †
Jepsen et al (2015)^35^	RCT	Total: 59 Patients/59 Implants G1: 33 Patients/33 Implants G2: 26 Patients/26 Implants	12 m	G1: 89.4% ± 20.7 G2: 85.8% ± 23.9	G1: 33.3% ± 31.7 G2: 40.4% ± 37.1	G1: 6.3 ± 1.3 G2: 6.3 ± 1.6	G1: 3.5 ± 1.5 G2: 3.5 ± 1.1	G1: 25.8% ± 36.8 G2: 21% ± 28.7	G1: 24.8% ± 36.3 G2: 10.3% ± 20	Mesial G1: 5.55 ± 2.3 G2: 4.63 ± 2.68 Distal G1: 5.41 ± 2.72 G2: 4.45 ± 2.23	Mesial G1: 1.98 ± 1.99 G2: 3.63 ± 2.34 Distal G1: 1.96 ± 1.95 G2: 3.63 ± 2.32	Mesial G1: 3.58 ± 2.05 G2: 0.96 ± 1.35 Distal G1: 3.45 ± 2.16 G2: 0.84 ± 1.14
Aghazadeh et al (2012)^29^	RCT	Total: 45 Patients/71 Implants G1: 22 Patients/34 Implants G2: 23 Patients/37 Implants	12 m	G1: 87.5% ± 20.1 G2: 79.4% ± 28.9	G1: 48.4% (SE 5.4) G2: 26.7% (SE 4.7)	G1: 6 ± 1.3 G2: 6.2 ± 1.4	G1: 3.8 (SE 0.2) G2: 3.3 (SE 0.2)	G1: 21.4% ± 25.4 G2: 13% ± 23.7	G1: 18.7% (SE 3.6) G2: 4.1% (SE 3.1)	NM	NM	G1: 0.2 (SE 0.3) G2: 1.1 (SE 0.3)
Schwarz et al (2012)^45^	RCT	Total: 24 Patients/26 Implants G1: 10 Patients/10 Implants G2: 14 Patients/14 Implants	7 y	G1: 96.6% ± 10.6 G2: 100%	G1: 41.6% ± 27.5 G2: 39.9% ± 26.6	G1: 4.9 ± 1.4 G2: 5.2 ± 1.5	G1: 3.2 ± 0.8 G2: 3.2 ± 0.4	G1: 0.5 ± 0.5 * G2: 0.7 ± 0.6 *	G1: 0.7 ± 1.1 G2: 1.1 ± 0.9	NM	NM	NM

BoP, bleeding on probing; PD, probing depth; PI, plaque index; BL, bone level; BG, bone gain; RCT, randomized clinical trial; SE, standard error; G1, group 1; G2, group 2; m, months; NM, not mentioned; y, year; * Silness-Löe plaque index is used; † Median is reported.

**Table 2:** Details of the interventions

**Author & Year**	**Intervention**				**Merging status**	**Complications**	**Conclusion**
	**Decontamination**	**Bone graft**	**Membrane**	**Postoperative care**			
Renvert et al (2021)^43^	Full-thickness flap + Ti curette + Rotary Ti brush + 3% H_2_O_2_ + saline	G1: DBBM G2: -	G1: NBCM G2: -	Azithromycin (500 mg on day 1 and 250 mg for 4 days) + Ibuprofen 400 mg + CHX	NM	G1: 1 patient lost to follow-up + 1 implant failure before 12 months G2: 2 implant failures before 12 months	Additional use of DBBM and NBCM resulted in significantly more defect fill than with surgical debridement alone. No other differences were found between the groups.
Emanuel et al (2020)^44^	Full-thickness flap + Granulation tissue removal + Implant surface decontaminated using ultrasonic, sonic, or hand instrument + saline	G1: D-PLEX_500_ G2: -	G1: - G2: -	NM	NM	G1: - G2: 2 implants were lost and removed during the follow-up period	D-PLEX_500 _showed promising results in enabling the healing of peri-implantitis lesions. The antibacterial component of the bone graft material might create favorable conditions that enable implant surface decontamination and soft and hard tissue healing.
Polymeri et al (2020)^38^	Full-thickness flap + Ti curette + 3% H_2_O_2_ + saline	G1: Bio-Oss xenograft G2: EndoBon xenograft	G1: - G2: -	Amoxicillin (500 mg 3 per day) + Metronidazole (500 mg 2 per day) + Paracetamol 500 mg + 0.12% CHX	Non-Submerged	One patient refused to attend the follow-up examinations	The application of xenograft EndoBon was shown to be non-inferior to xenograft Bio-Oss when used in reconstructive surgery of peri-implant osseous defects.
De Tapia et al (2019)^40^	0.12% CHX + Full-thickness flap + granulation tissue removal with curette and ultrasonic + implantoplasty + ↓ G1: Ti brush G2: Plastic ultrasonic scaler + 3% H_2_O_2_	Alloplastic graft consisting of hydroxyapatite/tricalcium phosphate	Collagen membrane	Amoxicillin (500 mg 3 per day) + Metronidazole (500mg 3 per day) + 0.12% CHX (twice daily)	Non-Submerged	G1: 1 membrane exposure G2: Two patients were lost during the follow-up period + 1 patient was excluded due to progressive bone loss and subsequent explantation	The additional use of a Ti brush during regenerative treatment of peri-implantitis resulted in statistically significant benefits in terms of PD reduction after 12 months.
Isler et al (2018)^37^	Full-thickness flap + granulation removal using Ti curette + saline	Bio-Oss xenograft	G1: CGF G2: Collagen membrane	Amoxicillin (500 mg 3 per day) + Metronidazole (500 mg 3 per day) + 0.12% CHX (twice a day) + Flurbiprofen (100 mg)	Submerged	G1: 3 patients refused to participate at follow-up + 1 implant showed suppuration and was removed G2: 2 patients refused to participate at follow-up + 3 implants showed slight membrane exposure	Both regenerative approaches yielded significant improvements in both clinical and radiographic assessments. The procedure using a collagen membrane in combination with a bone substitute showed better results at 12 months in regenerative treatment of peri-implantitis.
Isler et al (2018)^36^	Full-thickness flap + Ti curette + saline + ↓ G1: OzoneDTA G2: -	Bio-Oss xenograft mixed with CGF	CGF	Amoxicillin (500 mg 3 per day) + Metronidazole (500 mg 3 per day) + 0.12% CHX (twice a day) + Flurbiprofen (100 mg)	NM	G1: 2 patients left the study G2: 3 patients left the study	Implant surface decontamination with the additional use of ozone therapy in the regenerative treatment of peri-implantitis showed clinically and radiographically significant.
Renvert et al (2018)^42^	Surgical flap + Ti curette + 3% H_2_O_2_ + saline	G1: EndoBon Xenograft G2: -	G1: - G2: -	Zitromax (500 mg on day 1 and 250 mg for days 2-4) + Ibuprofen 400 mg + 0.2% CHX	NM	NM	Successful treatment outcomes using a bone substitute were more predictable when a composite therapeutic endpoint was considered.
Andersen et al (2017)^41^	Surgical open flap mechanical and chemical debridement with Ti curette and 24% EDTA gel	G1: PTG G2: -	G1: - G2: -	Amoxicillin (7 days) + Metronidazole (7 days)	Submerged	5 patients died + 10 patients lost to follow-up G1: 3 patients lost their treated implants + 2 patients excluded due to technical complications with supraconstructions + 1 patient received a new crown + 1 patient had an overdenture	This long-term follow-up of surgical treatment of peri-implant osseous defects showed unpredictable results.
Isehed et al (2016)^39^	Surgical flap + granulation tissue removal + ultrasonic + Ti instruments + saline	G1: Emdogain Enamel Matrix Derivative G2: -	G1: - G2: -	2 mg/ml CHX	NM	G1: 3 patients lost to follow-up (1 discontinued for personal reasons + 2 used systemic antibiotics following severe reinfection) G2: 1 implant disintegrated	Adjunctive Emdogain to surgical treatment of peri-implantitis was associated with prevalence of Gram + /aerobic bacteria during the follow-up period and increased marginal BL 12 months after treatment.
Jepsen et al (2015)^35^	Full-thickness flap + Ti curette + Ti brush + 3% H_2_O_2_ + saline	G1: PTG G2: -	G1: - G2: -	Amoxicillin (500 mg 3 per day) + Metronidazole (400 mg 2 per day) + Ibuprofen (600 mg 3 per day) + 0.2% CHX	Non-submerged	G2: 4 patients were lost to follow-up	Reconstructive surgery using PTGs resulted in significantly enhanced radiographic defect fill compared with open flap debridement. Similar improvements according to clinical measures were obtained after both surgical treatment modalities
Aghazadeh et al (2012)^29^	Full-thickness flap + Ti instruments + 3% H_2_O_2_ + saline	G1: Autogenous bone from mandibular ramus G2: Bio-Oss xenograft	Collagen membrane	Azithromycin (250 mg for 4 days) + Ibuprofen (400 mg) + 0.1% CHX	Non-submerged	No complication occurred	Bovine xenograft provided more radiographic bone fill than autogenous bone. The success of both surgical regenerative procedures was limited. Decreases in PD, BOP, and suppuration were observed.
Schwarz et al (2012)^45^	Full-thickness flap + Plastic curette + implantoplasty + saline + ↓ G1: Er:YAG laser G2: Plastic curette	Bio-Oss xenograft	Collagen membrane	0.2% CHX (twice a day)	Non-submerged	4 implants in the G1 and 8 implants in the G2 received additional peri-implantitis treatment at 24 months due to clinical signs suggesting reinfection	The long-term stability of clinical outcomes obtained following combined surgical therapy of advanced peri-implantitis may be influenced by factors other than the method of surface decontamination.

G1, group 1; G2, group 2; DBBM, demineralized bovine bone mineral; NBCM, native bilayer collagen membrane; CHX, chlorhexidine; NM, not mentioned; PD, probing depth; CGF, concentrated growth factor; PTG, porous titanium granule; BL, bone level; BoP, bleeding on probing.

**Table 3:** Summary of measurements from studies with follow-ups longer than 12 months

**Author & Year**	**Follow-up period**	**Clinical & radiographic parameters**				
		**BoP**	**PD**	**PI**	**BL**	**BG**
Isehed et al (2018)^46^	5 y	G1: 55.6% G2: 40%	NM	G1: 28.6% G2: 0%	G1: 4.1 † G2: 3.3 †	NM
Isehed et al (2018)^46^	3 y	G1: 80% G2: 62.5%	NM	G1: 20% G2: 33.3%	G1: 4.8 † G2: 3.8 †	NM
Andersen et al (2017)^41^	7 y	G1: 75% G2: 78%	NM	G1: 19.6% ± 15.5 G2: 28.8% ± 35.1	NM	NM
Schwarz et al (2016)^47^	7 y	G1: 6.6% ± 14.9 G2: 10% ± 11.65	G1: 4.04 ± 1.05 G2: 3.55 ± 1.3	G1: 0.32 ± 0.4 * G2: 0.62 ± 0.73 *	NM	NM
Schwarz et al (2013)^48^	4 y	G1: 23.5% ± 23.4 G2: 14.8% ± 16.4	G1: 3.8 ± 1.1 G2: 4.3 ± 1.2	G1: 0.8 ± 0.7 * G2: 0.8 ± 0.7 *	NM	NM
Schwarz et al (2012)^45^	2 y	G1: 21.6% ± 33.3 G2: 45.1% ± 30.4	G1: 3.8 ± 1.3 G2: 3.7 ± 1.1	G1: 0.3 ± 0.4 * G2: 0.7 ± 0.6 *	NM	NM

BoP, bleeding on probing; PD, probing depth; PI, plaque index; BL, bone level; BG, bone gain; y, year; G1, group 1; G2, group 2; NM, not mentioned. † Median is reported; * Silness-Löe plaque index is used.

###  Components of treatment

 Peri-implant bone defects were filled with various materials, including xenografts, autografts, alloplasts, growth factors, etc. (Table 2).^29,35-46^ Aghazadeh et al^29^ reported a greater bone fill through xenograft insertion compared to autograft. In another study, Polymeri et al^38^ found no significant difference between the two types of xenografts, namely EndoBon and Bio-Oss.

 In six studies, collagen or concentrated growth factor (CGF) membranes were used to cover the grafting materials.^29,36,37,40,43,45^ Isler et al^37^ compared collagen and CGF membranes for covering similar bone substitutes. The results demonstrated significant improvements with both modalities, but using collagen membranes resulted in superior outcomes. Among the seven studies mentioning the merging status following the treatment, five selected non-submerged healing,^29,35,38,40,45^ and two opted for submerged healing.^37,41^

 The decontamination phase of the treatments involved a combination of mechanical and chemical techniques. Mechanical methods included plastic curettes, Ti curettes/brushes, ultrasonic devices, sonic devices, and implantoplasty.^35-37,39-41,44,45^ Chemical agents such as saline, H_2_O_2_, NaCl, ozone, and EDTA were employed during chemical debridement.^29,35-45^ A comparison of decontamination methods was conducted in the study by De Tapia et al,^40^ which revealed that the additional use of a Ti brush resulted in a significant PD reduction. One study used an Er:YAG laser to decontaminate the peri-implant site.^45^ However, laser application failed to obtain significantly superior outcomes compared to conventional decontamination via plastic curettes. In terms of postoperative care, the most frequently prescribed medications included ibuprofen, amoxicillin, azithromycin, metronidazole, and CHX.^29,35-45^

 Of the six studies reporting bone gain or loss,^35,39,41-44^ one showed a deterioration of 1.9 mm,^41^ while another showed a maximum BG of 3.63 mm during the first year.^35^ The former study used porous Ti granules (PTG) as the bone substitute without membrane coverage, while the latter observed BG solely through curettage without graft or membrane materials.

###  Meta-analysis results

 After categorizing the comparisons made within the included studies, they were classified into:

- Using versus not using bone graft^35,39,41-44^
- Decontamination methods^36,40,45^
- Types of bone substitutes^29,38^
- Types of membranes covering the bone substitute^37^

**Figure 3:**
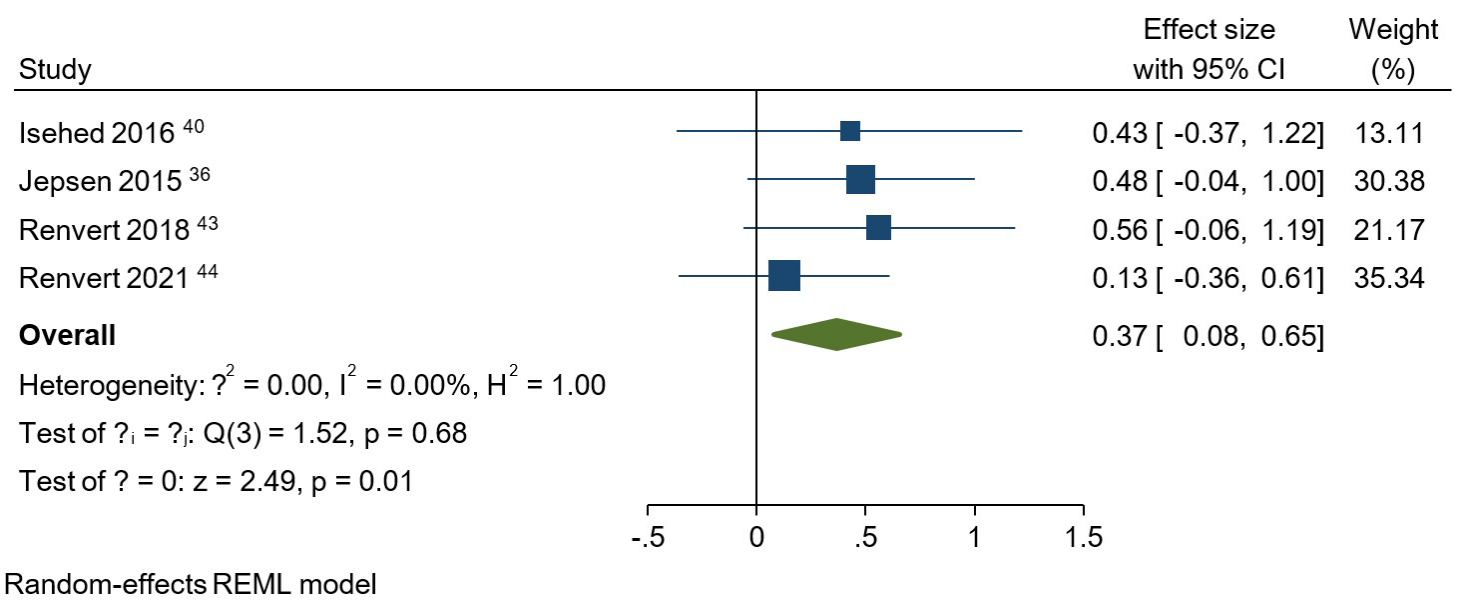
Meta-analysis for plaque index at the one-year follow-up among studies that compared using and not using bone grafts

**Figure 4:**
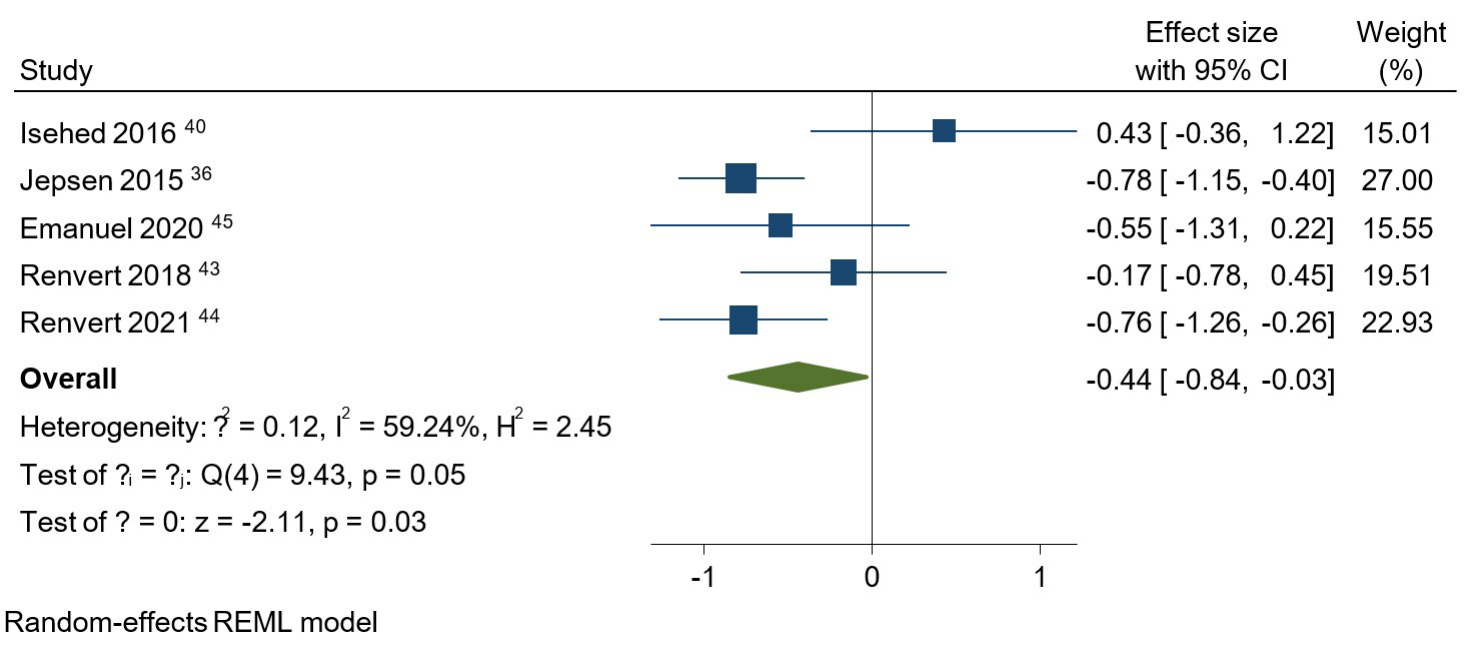
Meta-analysis for bone level at the one-year follow-up among studies that compared using and not using bone grafts

Due to the limited number of included studies and the diversity in the interventions they examined, only the six articles in the first category were sufficient to conduct a meta-analysis. Consequently, additional comparisons between different bone substitutes, membranes, healing status, and decontamination methods could not be established. The results of the meta-analyses for six parameters at baseline and one-year follow-up can be found in Figures 3 and 4 (Figures S1 to S7). Notably, the study by Jepsen et al^35^ reported each parameter separately for the mesial and distal aspects. Thus, a separate meta-analysis was conducted for this study to obtain a single value for each parameter and avoid biased weighting compared to the other studies.

 At baseline, no statistically significant difference was observed between the studies regarding BoP, PD, PI, or BL (Figures S1 to 4). Regarding BoP at the one-year follow-up, the overall effect size for implementing bone grafts was 0.04 (95% CI: -0.26‒0.35) (Figure S5). However, this intervention did not result in a significantly lower BoP compared to not using bone grafts. The PD analysis at the one-year follow-up indicated that using bone substitutes in regenerative treatments did not show a significant advantage over approaches without these materials (Figure S6). In this regard, the overall effect size was -0.08 (95% CI: -0.42‒0.27). An overall effect size of 0.37 (95% CI: 0.08‒0.65) was obtained for bone substitutes regarding PI at one year (Figure 3). It was concluded that using bone grafts, regardless of their type, significantly boosted the decrease in PI values (*P* = 0.01). When it came to BL comparison between the studies at one year (Figure 4), it was observed that using a bone graft during surgical regeneration had a significant positive impact on BL improvements, with an overall effect size of -0.44 (95% CI: -0.84 to -0.03). Similar to BoP and PD, bone grafts did not significantly influence the amount of BG following a one-year interval (Figure S7). The overall effect size equaled 0.16 (95% CI: -0.68‒1.01) for BG.

 The *P* values for the test of θ_i_ = θ_j_ were > 0.05 for all parameters at baseline, indicating that the studies were homogeneous at baseline. At the one-year follow-up, the BoP, PD, and PI analyses showed homogeneity, while the *P* values for BL and BG were ≤ 0.05 (*P* = 0.05 and *P* = 0.00, respectively), indicating that the studies were heterogeneous in terms of these two parameters.

###  Risk of bias assessment

 The risk of bias assessment revealed that five studies had a low risk of bias, and seven raised some concerns (Table 4). In addition, one study exhibited a high risk of bias and, as a consequence, was omitted due to exclusion criteria.

**Table 4:** Risk of bias assessment

**Author & Year**	**Randomization process**	**Deviations from intended interventions**	**Missing outcome data**	**Outcome measurement**	**Selection of reported result**	**Overall**
Renvert et al (2021) ^43^	+	+	+	+	+	Low
Emanuel et al (2020) ^44^	+	?	+	+	+	Some concerns
Polymeri et al (2020) ^38^	+	+	+	+	+	Low
De Tapia et al (2019) ^40^	+	+	+	+	+	Low
Isler et al (2018) ^37^	+	?	+	?	+	Some concerns
Isler et al (2018) ^36^	+	?	+	+	+	Some concerns
Renvert et al (2018) ^42^	+	+	+	+	+	Low
Andersen et al (2017) ^41^	+	?	+	+	?	Some concerns
Isehed et al (2016) ^39^	+	?	+	?	?	Some concerns
Jepsen et al (2015) ^35^	+	?	+	?	+	Some concerns
Aghazadeh et al. (2012) ^29^	+	+	+	?	+	Some concerns
Schwarz et al (2012) ^45^	+	+	+	+	+	Low

+ Low risk; - High risk; ? Some concerns.

## Discussion

###  Main findings

 This systematic review aimed to compare the clinical and radiographic outcomes of different regenerative protocols for peri-implantitis treatment. The findings revealed significant improvements in PI and BL one year after using bone grafts. However, using bone substitutes did not significantly affect the BoP, PD, and BG values. Various factors, including the decontamination method, postoperative care, and graft type, can also impact treatment outcomes alongside the surgical approach.

###  Bleeding on Probing

 Renvert et al.^43^ recorded the lowest BoP (8.3%) one year after peri-implantitis treatment. This favorable outcome was achieved through decontamination with 3% H_2_O_2_ and saline. Similarly, a significant decrease in BoP was recorded in the study by Leonhardt et al.^49^ after applying H_2_O_2_ for decontamination. In contrast, the highest BoP (83%) was observed in the study by Andersen et al.^41^ after using a Ti curette accompanied by 24% EDTA gel. EDTA does not possess antimicrobial properties per se, and the additional usage of other chemicals, such as CHX, has been suggested for improved decontamination.^50^ Ramanauskaite et al.^15^ reported that regenerative interventions alongside conventional peri-implantitis treatment did not significantly enhance BoP changes. Supporting this finding, Daugela et al.^31^ showed that a regenerative strategy could not improve BoP reduction significantly, whether a barrier membrane was used or not.

###  Probing Depth

 One year after peri-implantitis treatment, decontamination with 3% H_2_O_2_ and saline in conjunction with a xenograft as the bone substitute resulted in the most favorable PD (2.6 mm).^42^ In line with this finding, Roccuzzo et al.^51^ achieved significantly reduced PDs by using xenograft for bone substitution. However, their decontamination phase involved 24% EDTA and 1% CHX gels. On the contrary, Emanuel et al.^44^ reported the least favorable PD (5.43 mm) following chemical decontamination with ultrasonic and saline. Luengo et al.^52^ reported that ultrasonic decontamination yielded less favorable results than air-polishing or Ti brushes, particularly when cleaning the implant threads within the apical third. Based on our findings, the use of bone grafts did not significantly differ from treatment approaches without bone substitution in terms of PD changes. Consistently, Li et al.^53^ concluded that the additional use of bone grafts did not significantly alter the changes in PD. When comparing regenerative, resective, and access flap procedures, it was found that PD reductions were relatively similar.^18^ On the contrary, Ramanauskaite et al.^15^ observed a greater PD reduction in studies using regenerative techniques along with conventional peri-implantitis treatment.

###  Plaque Index

 At the one-year follow-up, the lowest PIs were observed in the studies by Isehed et al.^43^ (0%) and Isler et al.^36^ (0.22 via the Silness-Löe index). The former performed open flap debridement (OFD) using an ultrasonic device, Ti instruments, and saline irrigation. In the latter, decontamination was executed using Ti curette, saline, and ozone DTA, while the bone defect was filled with xenograft and CGF. In several studies, ozone therapy has been shown to improve PI and PD.^54-56^ McKenna et al.^57^ reported that ozone application significantly decreased PI in patients with peri-implant mucositis, which aligns with the results of Isler et al.^36^

 On the other hand, PI was the highest in the studies by Renvert et al.^42^ (25%) and Schwarz et al.^45^ (1.1 via the Silness-Löe index). The former executed chemomechanical decontamination using 3% H_2_O_2_, saline, and Ti curettes, while the latter used implantoplasty, saline, and plastic curettes for decontamination, as well as xenograft covered with collagen membrane for bone substitution. According to Monje et al.,^58^ incorporating implantoplasty into resective or reconstructive strategies of peri-implantitis treatment did not significantly improve clinical parameters, including PI. Furthermore, incorporating implantoplasty into regenerative treatment did not necessarily result in a marked amelioration in clinical measurements.^59^ Also, regenerative treatments may not necessarily be superior to non-regenerative methods in PI reduction. However, it should be remembered that employing a barrier membrane in regenerative protocols would give rise to significant PI enhancement.^18^

###  Bone Level

 The highest one-year BL change (3.57 mm) was obtained in a study by Jepsen et al.^35^ This change resulted from decontamination with Ti curette, 3% H_2_O_2_, and saline, as well as bone substitution with PTG. In another study, using PTGresulted in greater improvements in periodontal indices compared to a xenograft.^60^ The most undesirable BL change (-0.33 mm) occurred in a study by Emanuel et al.,^44^ in which ultrasonic and saline were used for decontamination, and D-PLEX_500_ was used to fill the bony defect. D-PLEX_500 _is a biodegradable, prolonged-release antibiotic-formulated bone graft that contains β-tricalcium phosphate granules coated with doxycycline hyclate. In contrast to the mentioned finding, De Tapia et al.^40^ concluded that implementing β-tricalcium phosphate as the bone substituting material would significantly enhance BL. Sanz-Martín et al.^18^ compared regenerative, resective, and access flap treatment methods and concluded that a regenerative approach could lead to more significant BL gains.

###  Bone Gain

 Jepsen et al.^35^ reported the highest BG at one year, with 3.58 mm in the control group. This study utilized OFD using a Ti curette, a Ti brush, 3% H_2_O_2_, and saline. On the contrary, the least favorable outcome (1.9 mm bone loss) was found in a study by Andersen et al.,^41^ following OFD with a Ti curette and EDTA gel and bone substitution using PTG. Conversely, Jepsen et al.^35^ concluded that employing PTG outperformed OFD regarding defect fill. Moreover, Guler et al.^60^ reported significant superiority for PTG over xenograft placement. It can be assumed that the undesirable outcomes in the study of Andersen et al.^41^ might be attributed to factors other than bone graft material.

 Based on our findings, bone grafts did not significantly affect the amount of BG. Another systematic review exploring various surgical regenerative treatments reported the greatest increase in marginal BL in three studies using enamel matrix derivative (EMD), platelet-derived growth factor (PDGF), bovine-derived xenograft, and PTG. Notably, xenografts and PTGs can appear radiopaque, making it difficult to distinguish them from regenerated bone.^31^ Overall, combining regenerative measures with conventional surgical peri-implantitis treatments would achieve greater defect fill.^15^ However, the complete resolution of a bony defect following guided bone regeneration (GBR) cannot be predicted with certainty.^61^

 Diverse materials, such as xenografts, autografts, etc., were used in the reviewed studies to fill bony defects. Some research outside this review combined bovine hydroxyapatite with nanocrystalline calcium sulfate, resulting in enhanced and stable outcomes.^62^ Mandelaris and DeGroot^63^ used a bone graft made of mineralized freeze-dried bone allograft (FDBA) and xenograft, paired with recombinant human platelet-derived growth factor (rhPDGF). Wen et al.^64^ combined FDBA and mineralized bovine and autogenous bone, proving their efficacy in peri-implantitis reconstructive procedures. The application of platelet-rich fibrin has also demonstrated successful resolution of bony defects.^65,66^ Kadkhodazadeh et al.^67^ successfully managed extensive peri-implant defects by employing a Ti mesh, autogenous bone, FDBA, and acellular dermal matrix. Augmentation in bone height and attachment level can be achieved by impregnating bone grafts with tetracycline, vancomycin, or tobramycin during the GBR of peri-implantitis-affected sites. Local application of antibiotics would be advantageous concerning the absence of side effects associated with systemic administration.^68,69^

###  Barrier Membranes

 After intrabony defect debridement, various types of cells can proliferate within the defect, including epithelial cells, connective tissue cells, bone cells, and periodontal ligament (PDL) cells. Barrier membranes can be employed to selectively allow bone cells to occupy the defect and provide physical stability for the bone substitute.^70^ Among the six studies using barrier membranes, collagen membranes and CGF were the two options.^29,36,37,40,43,45^ However, there is ongoing debate regarding the benefits of covering bone grafts with membranes. Isler et al.^37^ compared two different barrier membranes, CGF and collagen, along with the same bone substitute. Collagen membranes were reported to yield more satisfactory results at the one-year follow-up. Monje et al.^71^ depicted that adding a resorbable cross-linked barrier to allograft did not impact the results of defect filling. Chan et al.^72^ suggested that applying barrier membranes in conjunction with graft materials may enhance outcomes compared to grafts alone. In contrast, Daugela et al.^31^ showed that the additional use of barrier membranes did not significantly improve clinical outcomes. In essence, current knowledge does not necessarily support the superiority of using barrier membranes over not using them.

 Despite the previously mentioned materials for bone graft coverage, Dong et al.^73^ reported encouraging outcomes after applying a nanofiber barrier membrane made up of magnesium oxide as the antibacterial agent alongside parathyroid hormone as the pro-osteogenic drug. In a case report, a non-resorbable Ti-reinforced polytetrafluoroethylene membrane was coupled with an absorbable collagen membrane to achieve immobility in the reconstructed region and enhanced wound healing.^63^ Human amnion-chorion membranes tested for GBR showed promising results after peri-implantitis treatment.^74^

 Membrane exposure is a potential complication after GBR, reducing the success rate extensively.^75^ Garcia et al.^76^ noted that barrier exposure during the treatment of peri-implant defects would decrease healing chances by 27%. Alarmingly, human studies report exposure rates up to 87.6%.^72^ Despite their potential benefits, barrier membranes can be costly, time-consuming, and technically sensitive, which might not justify their use in specific configurations such as three-wall defects.^72^

###  Decontamination

 In addition to conventional decontamination techniques, the Er:YAG laser was applied in one study.^45^ At the 2- and 7-year follow-ups, laser-treated subjects did not exhibit significant differences in BoP or CAL reduction.^45,47^ However, plastic curette debridement demonstrated a significantly greater reduction in both BoP and CAL after four years.^48^ There is limited research regarding the advantages of using lasers to treat peri-implantitis. Chala et al.^77^ found that the benefits of applying lasers are confined to a short-term follow-up of three months. Even the short-term clinical benefits of the Er:YAG laser for surface decontamination were refuted in another study.^78^

 Additional use of ozone alongside saline has yielded improved clinical and radiographic outcomes.^36^ Ozone has also diminished bacterial adhesion to Ti and zirconia surfaces in vitro without inhibiting osteoblast proliferation.^79^ In brief, the techniques employed for implant decontamination did not significantly impact the results following surgical regenerative procedures, and none exhibited superiority over others.^78,80^ Additionally, the decontamination technique must be tailored to implant surface characteristics for optimal biofilm removal.^81^

###  Postoperative Care

 Antibiotics, namely amoxicillin and metronidazole, were the most common options. Although the prescription of these two antibiotics has been shown to be beneficial for peri-implantitis treatment,^78^ the efficacy of local or systemic administration of metronidazole remains unclear.^82^ CHX, an antiseptic, was widely used in almost all studies. The combination of azithromycin, ibuprofen, and CHX resulted in satisfactory periodontal improvements,^42,43^ while amoxicillin in conjunction with metronidazole was another proper choice for postoperative care.^35,38^ Overall, a personalized evaluation must be performed before the prescription of systemic antibiotics due to insufficient evidence supporting the integration of this drug delivery route into the standard treatment protocol.^83^

 Although some research favors non-submerged healing, a consensus report advocates for submerged healing, as it stimulates protected physiological wound closure.^32^ Keeping the suprastructure in place during surgical treatment may negatively affect the efficacy of postoperative oral hygiene maintenance, intraoperative decontamination, flap design, and numerical measurements.^32^ The non-submerged approach has also satisfied clinicians regarding clinical and radiographic improvements following peri-implantitis therapy.^84^ The debate continues since any relationship between the success of peri-implantitis treatment and the postoperative merging status has been refuted.^85^

###  Other Factors

 Regardless of the materials and techniques implemented throughout peri-implantitis treatment, other factors such as implant location, defect morphology, and implant surface characteristics can be differential.^21,36,86-88^ In addition to the higher prevalence of peri-implantitis within the upper jaw,^89^ maxillary implants are more responsive to regenerative treatments.^36^ Although Roccuzzo et al.^90^ reported no significant association between defect configuration and defect resolution, Aghazadeh et al.^86^ observed enhanced defect fill in four-wall and deeper defects. Also, Schwarz et al.^21^ found a higher likelihood of resolution for circumferential defects than for dehiscence-type defects. A review of animal studies highlighted the crucial role of surface characteristics in peri-implantitis progression and treatment outcomes as opposed to the onset of the disease. In detail, treated surfaces represented the minimum BL and most desirable outcomes.^87^ Furthermore, improvements were more pronounced around sandblasted and acid-etched implants than Ti plasma-sprayed implants after regenerative treatment.^88^ Re-osseointegration has also been reported to occur more frequently around smooth-surface implants than around moderately rough implants.^91^

 Peri-implantitis risk factors can be divided into five categories, including factors associated with the patient, implant design, implant site, prosthesis, and clinician.^92^ Achieving satisfactory long-term outcomes becomes possible when the primary cause is accurately identified and addressed. A 3% recurrence rate for peri-implantitis has been reported following surgical intervention, potentially resulting in a 36% implant loss in the long term.^93^ Factors such as deep residual PD, recessed marginal BL, and implant surface modification during surgical peri-implantitis treatment were identified as contributors to disease recurrence at a surgically treated site.^94^ Despite the limited number of histological examinations on re-osseointegration following regenerative treatment on previously contaminated implant surfaces,^95^ re-osseointegration seems feasible given that an effective decontamination method and suitable regenerative strategy are employed.^91,96^

 Some argue that peri-implantitis is more of a foreign-body reaction than a bacterial-triggered disease such as periodontitis, suggesting that peri-implant bone loss can be traced to an osteolytic immune reaction. As in most cases, a physiologic balance is often established between osteoblast and osteoclast activity, making the long-term survival of implants feasible. However, when other factors, such as genetic variations, smoking, excessive cement, bacterial contamination, and technical issues, are added to the foreign-body reaction, the equilibrium gets disrupted, leading to bone loss.^97,98^ Skeptics of this theory argue that there is insufficient evidence to solidify the pivotal role of foreign-body reactions in the pathogenesis of peri-implantitis. They assert that dental plaque biofilm is the principal causative agent of peri-implantitis, which should be the focus of both preventative and therapeutic measures.^99^

## Limitations

 The vast variability in peri-implantitis treatment components affected the reliability of inter-study comparisons and prevented the establishment of a standardized protocol. Differing disease definitions and outcomes may have also added heterogeneity. Soft tissue parameters and factors such as smoking or genetics were not addressed. Lastly, the full text of one study was unavailable, so data from its 7-year follow-up was used instead.^41^

## Conclusion

 Following the comparison between various surgical regenerative protocols in peri-implantitis treatment, it was concluded that employing bone grafts did not significantly improve the parameters of BoP, PD, and BG, yet PI and BL showed significant enhancements. Decontamination predominantly relied on Ti instruments and chemicals such as H_2_O_2_. A variety of bone substitutes, including xenografts and CGF, were employed. Approximately half of the studies utilized collagen or CGF membranes, while others opted for none. Postoperative care involved a mix of antibiotics, CHX, and analgesics. Given the diverse materials and peri-implantitis definitions, more standardized trials are needed to establish a standardized protocol.

## Consent for Publication

 Not applicable.

## Competing Interests

 The authors declare that they have no competing interests.

## Data Availability Statement

 The authors confirm that the data supporting the findings of this study are available within the article and its supplementary materials.

## Ethical Approval

 Not applicable.

## Supplementary Files

Supplementary file contains Figures S1-S7.

## References

[1] Pjetursson BE, Tan K, Lang NP, Brägger U, Egger M, Zwahlen M (2004). A systematic review of the survival and complication rates of fixed partial dentures (FPDs) after an observation period of at least 5 years. Clin Oral Implants Res.

[2] Peri-implant mucositis and peri-implantitis: a current understanding of their diagnoses and clinical implications. J Periodontol 2013;84(4):436-43. 10.1902/jop.2013.134001. 23537178

[3] Berglundh T, Armitage G, Araujo MG, Avila-Ortiz G, Blanco J, Camargo PM (2018). Peri-implant diseases and conditions: consensus report of workgroup 4 of the 2017 World Workshop on the Classification of Periodontal and Peri-Implant Diseases and Conditions. J Clin Periodontol.

[4] Derks J, Tomasi C (2015). Peri-implant health and disease A systematic review of current epidemiology. J Clin Periodontol.

[5] Lang NP, Berglundh T (2011). Periimplant diseases: where are we now?--Consensus of the Seventh European Workshop on Periodontology. J Clin Periodontol.

[6] Belibasakis GN (2014). Microbiological and immuno-pathological aspects of peri-implant diseases. Arch Oral Biol.

[7] Renvert S, Polyzois I (2015). Risk indicators for peri-implant mucositis: a systematic literature review. J Clin Periodontol.

[8] Dreyer H, Grischke J, Tiede C, Eberhard J, Schweitzer A, Toikkanen SE (2018). Epidemiology and risk factors of peri-implantitis: a systematic review. J Periodontal Res.

[9] Monje A, Aranda L, Diaz KT, Alarcón MA, Bagramian RA, Wang HL (2016). Impact of maintenance therapy for the prevention of peri-implant diseases: a systematic review and meta-analysis. J Dent Res.

[10] Monje A, Catena A, Borgnakke WS (2017). Association between diabetes mellitus/hyperglycaemia and peri-implant diseases: systematic review and meta-analysis. J Clin Periodontol.

[11] Ramanauskaite A, Daugela P, Faria de Almeida R, Saulacic N (2016). Surgical non-regenerative treatments for peri-implantitis: a systematic review. J Oral Maxillofac Res.

[12] Khoshkam V, Suárez-López Del Amo F, Monje A, Lin GH, Chan HL, Wang HL (2016). Long-term radiographic and clinical outcomes of regenerative approach for treating peri-implantitis: a systematic review and meta-analysis. Int J Oral Maxillofac Implants.

[13] Renvert S, Lessem J, Dahlén G, Lindahl C, Svensson M (2006). Topical minocycline microspheres versus topical chlorhexidine gel as an adjunct to mechanical debridement of incipient peri-implant infections: a randomized clinical trial. J Clin Periodontol.

[14] Wang H, Liu Y, Li W, Li W, Xu H, Niu G (2021). Microbiota in gingival crevicular fluid before and after mechanical debridement with antimicrobial photodynamic therapy in peri-implantitis. Front Cell Infect Microbiol.

[15] Ramanauskaite A, Fretwurst T, Schwarz F (2021). Efficacy of alternative or adjunctive measures to conventional non-surgical and surgical treatment of peri-implant mucositis and peri-implantitis: a systematic review and meta-analysis. Int J Implant Dent.

[16] van Winkelhoff AJ (2012). Antibiotics in the treatment of peri-implantitis. Eur J Oral Implantol.

[17] González Regueiro I, Martínez Rodriguez N, Barona Dorado C, Sanz-Sánchez I, Montero E, Ata-Ali J (2021). Surgical approach combining implantoplasty and reconstructive therapy with locally delivered antibiotic in the treatment of peri-implantitis: a prospective clinical case series. Clin Implant Dent Relat Res.

[18] Sanz-Martín I, Cha JK, Sanz-Sánchez I, Figuero E, Herrera D, Sanz M (2021). Changes in peri-implant soft tissue levels following surgical treatment of peri-implantitis: a systematic review and meta-analysis. Clin Oral Implants Res.

[19] Mordini L, Sun N, Chang N, De Guzman JP, Generali L, Consolo U (2021). Peri-implantitis regenerative therapy: a review. Biology (Basel).

[20] Smeets R, Henningsen A, Jung O, Heiland M, Hammächer C, Stein JM (2014). Definition, etiology, prevention and treatment of peri-implantitis--a review. Head Face Med.

[21] Schwarz F, Sahm N, Schwarz K, Becker J (2010). Impact of defect configuration on the clinical outcome following surgical regenerative therapy of peri-implantitis. J Clin Periodontol.

[22] Jepsen S, Schwarz F, Cordaro L, Derks J, Hämmerle CHF, Heitz-Mayfield LJ (2019). Regeneration of alveolar ridge defects Consensus report of group 4 of the 15th European Workshop on Periodontology on Bone Regeneration. J Clin Periodontol.

[23] Greenstein G, Cavallaro J (2014). Failed dental implants: diagnosis, removal and survival of reimplantations. J Am Dent Assoc.

[24] Renvert S, Polyzois I, Claffey N (2012). Surgical therapy for the control of peri-implantitis. Clin Oral Implants Res.

[25] Almohandes A, Abrahamsson I, Dionigi C, Berglundh T (2022). Surgical treatment of experimental peri-implantitis using mechanical and chemical decontamination procedures: a pre-clinical in vivo study. J Clin Periodontol.

[26] Tan NCP Miller CM, Antunes E, Sharma D (2022). Impact of physical decontamination methods on zirconia implant surface and subsequent bacterial adhesion: an in-vitro study. Clin Exp Dent Res.

[27] Cai Z, Li Y, Wang Y, Chen S, Jiang S, Ge H (2019). Antimicrobial effects of photodynamic therapy with antiseptics on Staphylococcus aureus biofilm on titanium surface. Photodiagnosis Photodyn Ther.

[28] Clem D, Gunsolley JC (2019). Peri-implantitis treatment using Er:YAG laser and bone grafting A prospective consecutive case series evaluation: 1-year posttherapy. Int J Periodontics Restorative Dent.

[29] Aghazadeh A, Rutger Persson G, Renvert S (2012). A single-centre randomized controlled clinical trial on the adjunct treatment of intra-bony defects with autogenous bone or a xenograft: results after 12 months. J Clin Periodontol.

[30] Khoury F, Buchmann R (2001). Surgical therapy of peri-implant disease: a 3-year follow-up study of cases treated with 3 different techniques of bone regeneration. J Periodontol.

[31] Daugela P, Cicciù M, Saulacic N (2016). Surgical regenerative treatments for peri-implantitis: meta-analysis of recent findings in a systematic literature review. J Oral Maxillofac Res.

[32] Khoury F, Keeve PL, Ramanauskaite A, Schwarz F, Koo KT, Sculean A (2019). Surgical treatment of peri-implantitis - consensus report of working group 4. Int Dent J.

[33] Page MJ, Moher D, Bossuyt PM, Boutron I, Hoffmann TC, Mulrow CD (2021). PRISMA 2020 explanation and elaboration: updated guidance and exemplars for reporting systematic reviews. BMJ.

[34] Sterne JAC, Savović J, Page MJ, Elbers RG, Blencowe NS, Boutron I (2019). RoB 2: a revised tool for assessing risk of bias in randomised trials. BMJ.

[35] Jepsen K, Jepsen S, Laine ML, Anssari Moin D, Pilloni A, Zeza B (2016). Reconstruction of peri-implant osseous defects: a multicenter randomized trial. J Dent Res.

[36] Isler SC, Unsal B, Soysal F, Ozcan G, Peker E, Karaca IR (2018). The effects of ozone therapy as an adjunct to the surgical treatment of peri-implantitis. J Periodontal Implant Sci.

[37] Isler SC, Soysal F, Ceyhanlı T, Bakırarar B, Unsal B (2018). Regenerative surgical treatment of peri-implantitis using either a collagen membrane or concentrated growth factor: a 12-month randomized clinical trial. Clin Implant Dent Relat Res.

[38] Polymeri A, Anssari-Moin D, van der Horst J, Wismeijer D, Laine ML, Loos BG (2020). Surgical treatment of peri-implantitis defects with two different xenograft granules: a randomized clinical pilot study. Clin Oral Implants Res.

[39] Isehed C, Holmlund A, Renvert S, Svenson B, Johansson I, Lundberg P (2016). Effectiveness of enamel matrix derivative on the clinical and microbiological outcomes following surgical regenerative treatment of peri-implantitis A randomized controlled trial. J Clin Periodontol.

[40] de Tapia B, Valles C, Ribeiro-Amaral T, Mor C, Herrera D, Sanz M (2019). The adjunctive effect of a titanium brush in implant surface decontamination at peri-implantitis surgical regenerative interventions: a randomized controlled clinical trial. J Clin Periodontol.

[41] Andersen H, Aass AM, Wohlfahrt JC (2017). Porous titanium granules in the treatment of peri-implant osseous defects-a 7-year follow-up study. Int J Implant Dent.

[42] Renvert S, Roos-Jansåker AM, Persson GR (2018). Surgical treatment of peri-implantitis lesions with or without the use of a bone substitute-a randomized clinical trial. J Clin Periodontol.

[43] Renvert S, Giovannoli JL, Roos-Jansåker AM, Rinke S (2021). Surgical treatment of peri-implantitis with or without a deproteinized bovine bone mineral and a native bilayer collagen membrane: a randomized clinical trial. J Clin Periodontol.

[44] Emanuel N, Machtei EE, Reichart M, Shapira L (2020). D-PLEX500: a local biodegradable prolonged release doxycycline-formulated bone graft for the treatment for peri-implantitis A randomized controlled clinical study. Quintessence Int.

[45] Schwarz F, John G, Mainusch S, Sahm N, Becker J (2012). Combined surgical therapy of peri-implantitis evaluating two methods of surface debridement and decontamination A two-year clinical follow up report. J Clin Periodontol.

[46] Leonhardt A, Dahlén G, Renvert S (2003). Five-year clinical, microbiological, and radiological outcome following treatment of peri-implantitis in man. J Periodontol.

[47] Monje A, Amerio E, Cha JK, Kotsakis G, Pons R, Renvert S (2022). Strategies for implant surface decontamination in peri-implantitis therapy. Int J Oral Implantol (Berl).

[48] Roccuzzo M, Bonino F, Bonino L, Dalmasso P (2011). Surgical therapy of peri-implantitis lesions by means of a bovine-derived xenograft: comparative results of a prospective study on two different implant surfaces. J Clin Periodontol.

[49] Luengo F, Sanz-Esporrín J, Noguerol F, Sanz-Martín I, Sanz-Sánchez I, Sanz M (2022). In vitro effect of different implant decontamination methods in three intraosseous defect configurations. Clin Oral Implants Res.

[50] Li ZB, Li K, Du M, Ren SB, Yu Y (2023). Surgical treatment of peri-implantitis with or without adjunctive graft material: a systematic review and meta-analysis of randomized controlled trials. Int J Oral Maxillofac Surg.

[51] Al Habashneh R, Alsalman W, Khader Y (2015). Ozone as an adjunct to conventional nonsurgical therapy in chronic periodontitis: a randomized controlled clinical trial. J Periodontal Res.

[52] Skurska A, Pietruska MD, Paniczko-Drężek A, Dolińska E, Zelazowska-Rutkowska B, Zak J (2010). Evaluation of the influence of ozonotherapy on the clinical parameters and MMP levels in patients with chronic and aggressive periodontitis. Adv Med Sci.

[53] Hayakumo S, Arakawa S, Mano Y, Izumi Y (2013). Clinical and microbiological effects of ozone nano-bubble water irrigation as an adjunct to mechanical subgingival debridement in periodontitis patients in a randomized controlled trial. Clin Oral Investig.

[54] McKenna DF, Borzabadi-Farahani A, Lynch E (2013). The effect of subgingival ozone and/or hydrogen peroxide on the development of peri-implant mucositis: a double-blind randomized controlled trial. Int J Oral Maxillofac Implants.

[55] Monje A, Pons R, Amerio E, Wang HL, Nart J (2022). Resolution of peri-implantitis by means of implantoplasty as adjunct to surgical therapy: a retrospective study. J Periodontol.

[56] Lin CY, Chen Z, Chiang HL, Pan WL, Wang HL (2022). The impact of implantoplasty in regenerated and nonregenerated treatment modalities in peri-implantitis: a systematic review and meta-analysis. Int J Oral Maxillofac Implants.

[57] Guler B, Uraz A, Yalım M, Bozkaya S (2017). The comparison of porous titanium granule and xenograft in the surgical treatment of peri-implantitis: a prospective clinical Study. Clin Implant Dent Relat Res.

[58] Sahrmann P, Attin T, Schmidlin PR (2011). Regenerative treatment of peri-implantitis using bone substitutes and membrane: a systematic review. Clin Implant Dent Relat Res.

[59] Sallé MR, Deluiz D, Fletcher P, Santoro MF, Tinoco EM (2023). Decontamination and repair protocol promotes positive outcomes in implants affected by peri-implantitis: a human case series. Int J Periodontics Restorative Dent.

[60] Mandelaris GA, DeGroot B (2022). Bone construction surgery: a case report using recombinant human platelet-derived growth factor-BB. Clin Adv Periodontics.

[61] Wen SC, Barootchi S, Huang WX, Wang HL (2022). Surgical reconstructive treatment for infraosseous peri-implantitis defects with a submerged healing approach: a prospective controlled study. J Periodontol.

[62] Hamzacebi B, Oduncuoglu B, Alaaddinoglu EE (2015). Treatment of peri-implant bone defects with platelet-rich fibrin. Int J Periodontics Restorative Dent.

[63] Sun G, Cao L, Li H (2021). Effects of platelet-rich fibrin combined with guided bone regeneration in the reconstruction of peri-implantitis bone defect. Am J Transl Res.

[64] Kadkhodazadeh M, Amid R, Moscowchi A (2021). Management of extensive peri-implant defects with titanium meshes. Oral Maxillofac Surg.

[65] Shrivastava PK, Mahmood A, Datta S, Sengar P, Sybil D (2022). Tetracycline impregnated bone grafts in the management of peri-implantitis and guided bone regeneration around dental implants: a systematic review. Saudi Dent J.

[66] Nart J, de Tapia B, Pujol À, Pascual A, Valles C (2018). Vancomycin and tobramycin impregnated mineralized allograft for the surgical regenerative treatment of peri-implantitis: a 1-year follow-up case series. Clin Oral Investig.

[67] Larsson L, Decker AM, Nibali L, Pilipchuk SP, Berglundh T, Giannobile WV (2016). Regenerative medicine for periodontal and peri-implant diseases. J Dent Res.

[68] Monje A, Pons R, Vilarrasa J, Nart J, Wang HL (2023). Significance of barrier membrane on the reconstructive therapy of peri-implantitis: a randomized controlled trial. J Periodontol.

[69] Chan HL, Lin GH, Suarez F, MacEachern M, Wang HL (2014). Surgical management of peri-implantitis: a systematic review and meta-analysis of treatment outcomes. J Periodontol.

[70] Dong Y, Yao L, Cai L, Jin M, Forouzanfar T, Wu L (2023). Antimicrobial and pro-osteogenic coaxially electrospun magnesium oxide nanoparticles-polycaprolactone /parathyroid hormone-polycaprolactone composite barrier membrane for guided bone regeneration. Int J Nanomedicine.

[71] Bhide VM, Goldberg MB, Tenenbaum HC (2022). Surgical treatment of peri-implantitis with guided bone regeneration using dehydrated amnion-chorion membranes: a case report with a 2-year follow-up. Int J Periodontics Restorative Dent.

[72] Simion M, Baldoni M, Rossi P, Zaffe D (1994). A comparative study of the effectiveness of e-PTFE membranes with and without early exposure during the healing period. Int J Periodontics Restorative Dent.

[73] Garcia J, Dodge A, Luepke P, Wang HL, Kapila Y, Lin GH (2018). Effect of membrane exposure on guided bone regeneration: a systematic review and meta-analysis. Clin Oral Implants Res.

[74] Schwarz F, John G, Schmucker A, Sahm N, Becker J (2017). Combined surgical therapy of advanced peri-implantitis evaluating two methods of surface decontamination: a 7-year follow-up observation. J Clin Periodontol.

[75] Schwarz F, Hegewald A, John G, Sahm N, Becker J (2013). Four-year follow-up of combined surgical therapy of advanced peri-implantitis evaluating two methods of surface decontamination. J Clin Periodontol.

[76] Chala M, Anagnostaki E, Mylona V, Chalas A, Parker S, Lynch E (2020). Adjunctive use of lasers in peri-implant mucositis and peri-implantitis treatment: a systematic review. Dent J (Basel).

[77] Baima G, Citterio F, Romandini M, Romano F, Mariani GM, Buduneli N (2022). Surface decontamination protocols for surgical treatment of peri-implantitis: a systematic review with meta-analysis. Clin Oral Implants Res.

[78] Hauser-Gerspach I, Vadaszan J, Deronjic I, Gass C, Meyer J, Dard M (2012). Influence of gaseous ozone in peri-implantitis: bactericidal efficacy and cellular response An in vitro study using titanium and zirconia. Clin Oral Investig.

[79] Ramanauskaite A, Obreja K, Sader R, Khoury F, Romanos G, Koo KT (2019). Surgical treatment of periimplantitis with augmentative techniques. Implant Dent.

[80] Kubasiewicz-Ross P, Fleischer M, Pitułaj A, Hadzik J, Nawrot-Hadzik I, Bortkiewicz O (2020). Evaluation of the three methods of bacterial decontamination on implants with three different surfaces. Adv Clin Exp Med.

[81] López-Valverde N, López-Valverde A, Blanco-Rueda JA (2023). Efficacy of adjuvant metronidazole therapy on peri-implantitis: a systematic review and meta-analysis of randomized clinical studies. Front Cell Infect Microbiol.

[82] Øen M, Leknes KN, Lund B, Bunæs DF (2021). The efficacy of systemic antibiotics as an adjunct to surgical treatment of peri-implantitis: a systematic review. BMC Oral Health.

[83] Wen SC, Barootchi S, Wang HL, Huang WX (2022). Non-submerged reconstructive approach for peri-implantitis osseous defect with removal of implant crowns: one-year outcomes of a prospective case series study. J Periodontol.

[84] Astolfi V, Gómez-Menchero A, Ríos-Santos JV, Bullón P, Galeote F, Ríos-Carrasco B (2021). Influence of removing or leaving the prosthesis after regenerative surgery in peri-implant defects: retrospective study: 32 clinical cases with 2 to 8 years of follow-up. Int J Environ Res Public Health.

[85] Aghazadeh A, Persson RG, Renvert S (2020). Impact of bone defect morphology on the outcome of reconstructive treatment of peri-implantitis. Int J Implant Dent.

[86] Garaicoa-Pazmino C, Lin GH, Alkandery A, Parra-Carrasquer C, Suárez-López Del Amo F (2021). Influence of implant surface characteristics on the initiation, progression and treatment outcomes of peri-implantitis: a systematic review and meta-analysis based on animal model studies. Int J Oral Implantol (Berl).

[87] Roccuzzo M, Pittoni D, Roccuzzo A, Charrier L, Dalmasso P (2017). Surgical treatment of peri-implantitis intrabony lesions by means of deproteinized bovine bone mineral with 10% collagen: 7-year-results. Clin Oral Implants Res.

[88] Moraschini V, Kischinhevsky ICC, Sartoretto SC, de Almeida Barros Mourão CF, Sculean A, Calasans-Maia MD (2022). Does implant location influence the risk of peri-implantitis?. Periodontol 2000.

[89] Roccuzzo M, Mirra D, Pittoni D, Ramieri G, Roccuzzo A (2021). Reconstructive treatment of peri-implantitis infrabony defects of various configurations: 5-year survival and success. Clin Oral Implants Res.

[90] Almohandes A, Carcuac O, Abrahamsson I, Lund H, Berglundh T (2019). Re-osseointegration following reconstructive surgical therapy of experimental peri-implantitis A pre-clinical in vivo study. Clin Oral Implants Res.

[91] Fu JH, Wang HL (2020). Breaking the wave of peri-implantitis. Periodontol 2000.

[92] Martins OP, Baptista IP, Caramelo FJ (2021). Disease recurrence after surgical treatment of peri-implantitis—systematic review and meta-analysis. Front Oral Maxillofac Med.

[93] Carcuac O, Derks J, Abrahamsson I, Wennström JL, Berglundh T (2020). Risk for recurrence of disease following surgical therapy of peri-implantitis-a prospective longitudinal study. Clin Oral Implants Res.

[94] Schwarz F, Jepsen S, Obreja K, Galarraga-Vinueza ME, Ramanauskaite A (2022). Surgical therapy of peri-implantitis. Periodontol 2000.

[95] Koo KT, Khoury F, Keeve PL, Schwarz F, Ramanauskaite A, Sculean A (2019). Implant surface decontamination by surgical treatment of periimplantitis: a literature review. Implant Dent.

[96] Albrektsson T, Canullo L, Cochran D, De Bruyn H (2016). “Peri-implantitis”: a complication of a foreign body or a man-made “disease” Facts and fiction. Clin Implant Dent Relat Res.

[97] Albrektsson T, Dahlin C, Jemt T, Sennerby L, Turri A, Wennerberg A (2014). Is marginal bone loss around oral implants the result of a provoked foreign body reaction?. Clin Implant Dent Relat Res.

[98] Ivanovski S, Bartold PM, Huang YS (2022). The role of foreign body response in peri-implantitis: what is the evidence?. Periodontol 2000.

[99] Isehed C, Svenson B, Lundberg P, Holmlund A (2018). Surgical treatment of peri-implantitis using enamel matrix derivative, an RCT: 3- and 5-year follow-up. J Clin Periodontol.

