# Supplementary material for: Surgical regenerative methods for peri-implantitis treatment: A systematic review and meta-analysis
Source: J Adv Periodontol Implant Dent. 2024 Jul 31;16(2):144–59. doi: 10.34172/japid.2024.013 (PMC11699266; doi:10.34172/japid.2024.013)
Supplement: Supplementary file 1 — Supplementary file contains Figures S1-S7. [file japid-16-144-s001.pdf]

Shahbazi et al, *J Adv Periodontol Implant Dent,* 2024, 16(2), 144-S1
doi: 10.34172/japid.2024.013
https://japid.tbzmed.ac.ir

Supplementary Files

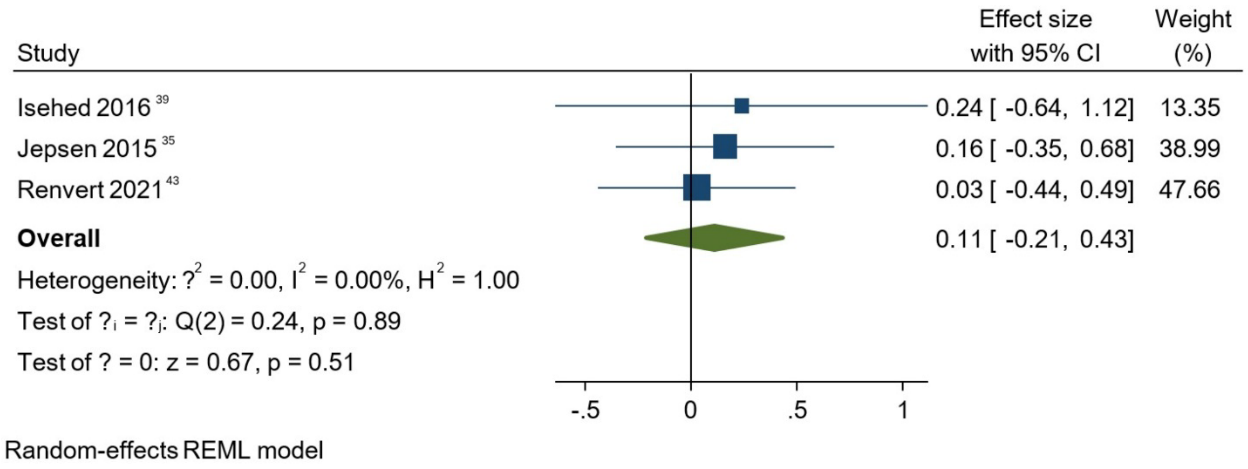

Figure S1. Meta-analysis for bleeding on probing at baseline among studies that compared using and not using bone grafts.

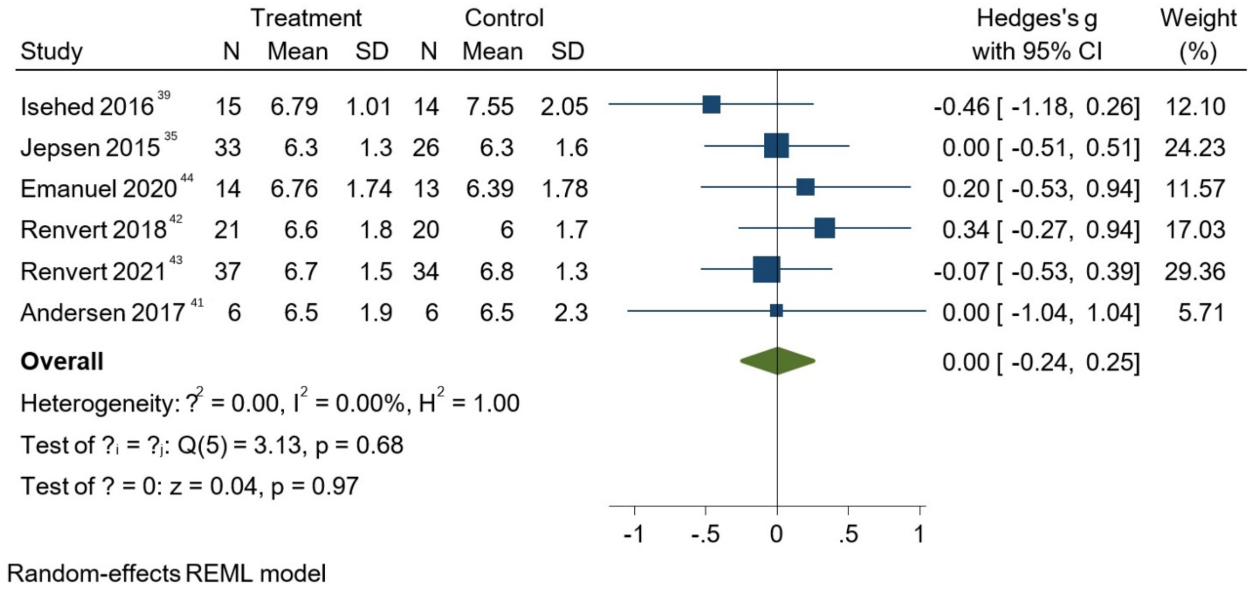

Figure S2. Meta-analysis for probing depth at baseline among studies that compared using and not using bone grafts.

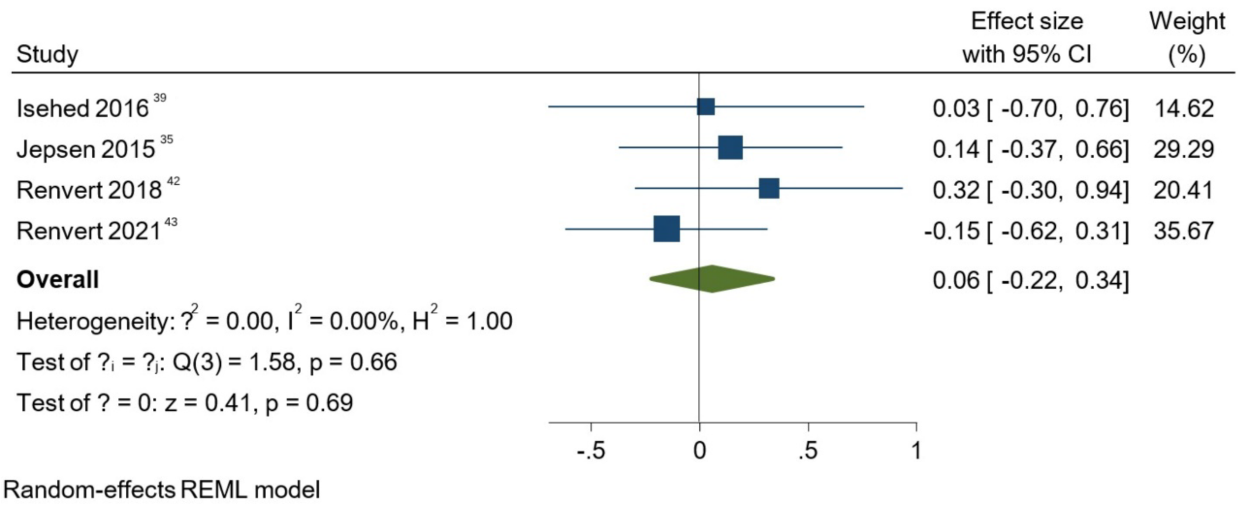

Figure S3. Meta-analysis for plaque index at baseline among studies that compared using and not using bone grafts.

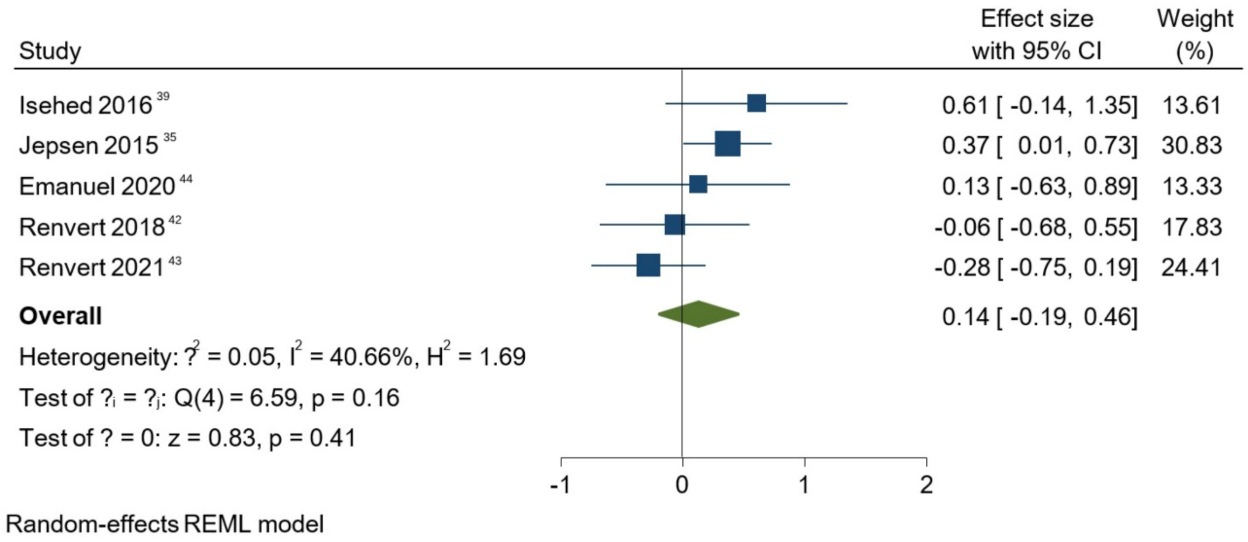

Figure S4. Meta-analysis for bone level at baseline among studies that compared using and not using bone grafts.

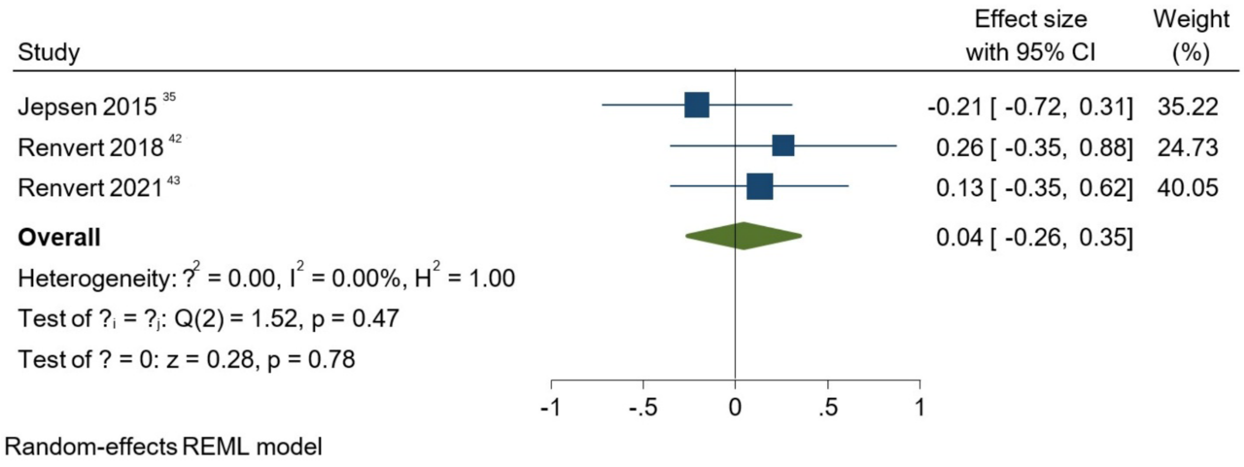

Figure S5. Meta-analysis for bleeding on probing at the one-year follow-up among studies that compared using and not using bone grafts.

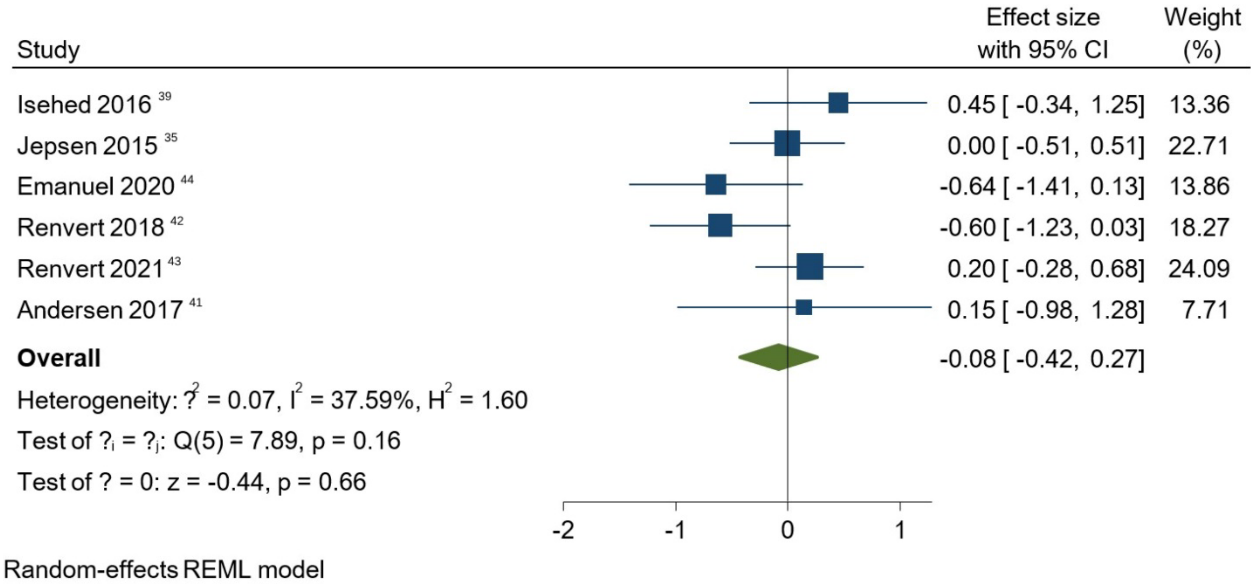

Figure S6. Meta-analysis for probing depth at the one-year follow-up among studies that compared using and not using bone grafts.

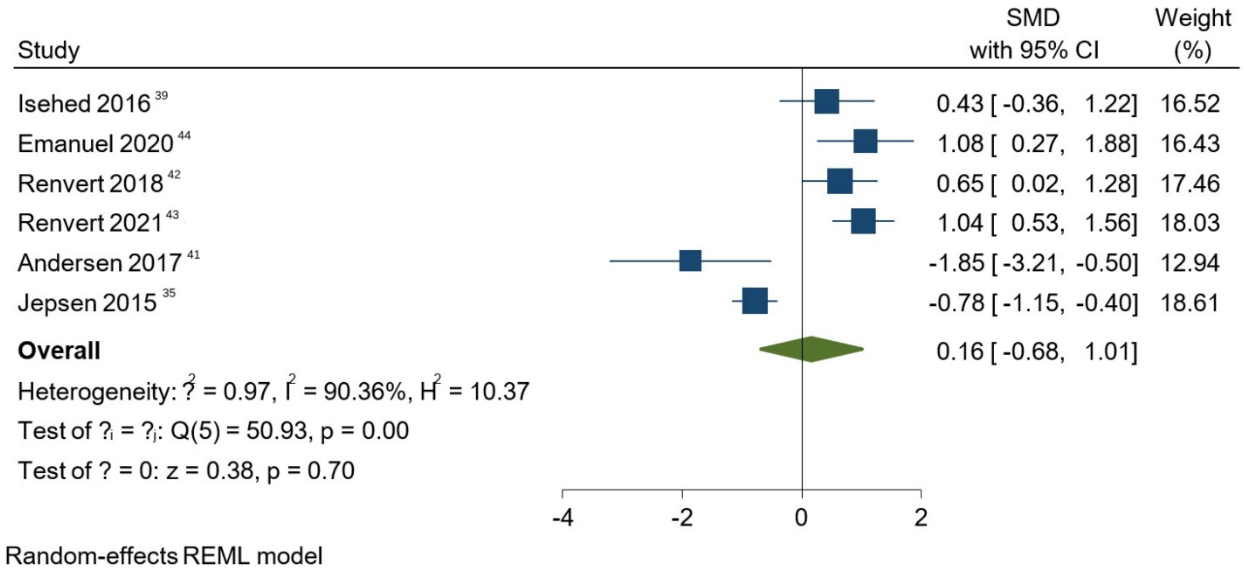

Figure S7. Meta-analysis for bone gain at the one-year follow-up among studies that compared using and not using bone grafts.
